# Supplementary material for: MetaRibo-Seq measures translation in microbiomes
Source: Nat Commun. 2020 Jun 29;11:3268. doi: 10.1038/s41467-020-17081-z (PMC7324362; doi:10.1038/s41467-020-17081-z)
Supplement: Supplementary file 10 — Supplementary Data 7 [file 41467_2020_17081_MOESM10_ESM.zip › File2/Confidence_High_Taxonomy/32994_out.krona.html]

Javascript must be enabled to view this page.

members
magnitude
magnitudeUnassigned
count
unassigned
taxon
rank

32994\_out

3

3
2
superkingdom

3
phylum
1239

3
186801
class

3
2044939

SRS014235\_contig\_number\_contig-100\_24104.66106SRS018836\_contig\_number\_contig-100\_16744.16745SRS142599\_contig\_number\_13300
species
